# Lung Cancer Incidence After September 11, 2001, Among World Trade Center Responders

**DOI:** 10.1001/jamanetworkopen.2025.36655

**Published:** 2025-10-09

**Authors:** Sean A. P. Clouston, Jaymie Meliker, Frank D. Mann, Pei-Fen Kuan, Yuan Yang, F. Mehejabin Trina, Laura Sampson, Paolo Boffetta, Norman H. Edelman, Benjamin J. Luft

**Affiliations:** 1 Program in Public Health, Stony Brook University, Stony Brook, New York; 2 Department of Family, Population, and Preventive Medicine, Renaissance School of Medicine at Stony Brook University, Stony Brook, New York; 3 Department of Medicine, Renaissance School of Medicine at Stony Brook University, Stony Brook, New York; 4 Department of Applied Mathematics & Statistics, Stony Brook University, Stony Brook, New York; 5 World Trade Center Health Program, Commack, New York

## Abstract

**Importance:**

Responders involved in rescue and recovery operations after the collapse of the World Trade Center (WTC) on September 11, 2001, were exposed to airborne carcinogens.

**Objectives:**

To examine the incidence of lung cancer after the WTC attacks and to compare the incidence of lung cancer among responders with varying degrees of exposure severity.

**Design, Setting, and Participants:**

In this prospective cohort study, data were collected between July 1, 2012, and December 31, 2023, from individuals who were enrolled in a medical monitoring program available to WTC responders residing on Long Island, New York. This study was restricted to people who survived and were followed up for incident lung cancer after a 10-year latency period.

**Exposures:**

Types and durations of exposures were based on responses to a detailed questionnaire about on-site work conditions, which included information about the type and duration of work, smells, and sights while working; exposure to dust; and the use of protective equipment. World Trade Center exposure characteristics and overall severity were measured as mild, moderate, and severe exposure using a validated approach.

**Main Outcomes and Measures:**

The incidence of lung cancer was the primary outcome. Diagnosis of lung cancer was ascertained following a standardized approach by trained clinicians, and diagnoses were verified by clinicians at the Centers for Disease Control and Prevention. Cox proportional hazards regression was used to estimate multivariable-adjusted hazard ratios.

**Result:**

Among 12 334 eligible responders (mean [SD] age at study inclusion, 49.3 [10.2] years; 11 213 men [90.9%]), 118 incident lung cancers were identified between July 1, 2012, and December 31, 2023 (incidence rate, 8.7/10 000 person-years [95% CI, 7.3-10.5 person-years]). When compared with mild exposures, the incidence of lung cancer was higher among moderately (adjusted hazard ratio [AHR], 1.86 [95% CI, 1.19-2.91]; *P* = .007) and severely (AHR, 2.90 [95% CI, 1.69-4.99]; *P* < .001) exposed groups. Specific WTC exposures, including smelling fumes (AHR, 1.05 [95% CI, 1.01-1.09]; *P* = .007) or sewage (AHR, 1.03 [95% CI, 1.01-1.05]; *P* = .004), were also associated with higher incidence of lung cancer after adjusting for demographics and measures of tobacco use.

**Conclusions and Relevance:**

In this cohort study of WTC responders, the incidence of lung cancer was higher among those with greater exposure severity. Future studies may investigate specific WTC exposures and histologic changes and clarify the role of WTC exposure for prognosis.

## Key Points

**Question:**

Is there an association between occupational exposures while working at the World Trade Center (WTC) disaster sites and the incidence of lung cancer over a decade later?

**Findings:**

In this cohort study including 12 334 WTC responders aged 49.3 years at study inclusion, 118 developed incident lung cancer more than a decade after exposure. Compared with minimally exposed responders who reported low dust exposure, responders working on the WTC sites who reported more severe exposures had a nearly 3-fold higher incidence of lung cancer even after adjusting for demographic factors and smoking.

**Meaning:**

This study suggests that, when compared with the lowest reported exposure levels, a higher level of reported exposure to more particulate dust or debris was significantly associated with an increased incidence of lung cancer.

## Teaser

This cohort study compares the incidence of lung cancer among responders to the September 11, 2001, attack on the World Trade Center with varying degrees of exposure severity and use of personal protective equipment.

## Introduction

On September 11, 2001, terrorist attacks caused the collapse of the World Trade Center (WTC), exposing thousands of individuals to a poorly characterized mix of toxicants.^1^ During the pursuant search, rescue, recovery, and cleanup efforts, many first responders, construction workers, and volunteers (hereafter, *responders*) were exposed to a wide array of toxic substances that varied by place and time. Researchers and clinicians hypothesized that exposures to these toxic substances may have increased the risk of lung cancer, but to date we have not identified an elevated risk of lung cancer among police officers and volunteer responders,^2^ firefighters,^3^ or other affected populations.^4^ Nevertheless, studies of acute and chronic respiratory symptoms have shown declines in respiratory functioning from preexposure levels,^5,6^ and abnormalities have been reported based on lung oscillometry^7^ and chest imaging.^8^ The potential for WTC exposures to be associated with an increased risk of lung cancer, therefore, remains a key concern for WTC responders and their families.^9^

Researchers have not identified a significant elevation in the risk of lung cancer among responders and survivors when compared with population statistics.^10^ However, the risk of lung cancer among responders should be lower, because only 9.1% of responders smoke now vs 12.9% of males residing in New York state in 2022.^11,12^ In addition, prior work has focused on the incidence of lung cancer in the first decade after exposure, a period that may fall outside the latency window for lung cancer development.^9^ Furthermore, efforts within the cohort have focused on exposure to the dust cloud generated by the collapse of the towers as the primary source of pulmonologic exposures.^13^ However, while the traumatic exposures on the first few days are highly memorable, many responders spent months digging through settled dust while pulverizing, burning, and aerosolizing it during recovery and cleanup operations.^14^

To address these limitations, we examined the incidence of lung cancer after 2012 in a cohort of WTC responders whose exposure to the WTC sites was heterogeneous, making assessment difficult. We hypothesized that a novel activities-based measure,^15^ developed to measure severity of exposure to fine particulate matter and airborne chemical pollutants, would find that exposure to the WTC sites was associated with the incidence of lung cancer more than 10 years after response operations ceased.

## Methods

The Committee on Research Involving Human Participants at Stony Brook University approved this study. Responders provided informed written consent; all procedures were completed following the protocol. We followed the Strengthening the Reporting of Observational Studies in Epidemiology (STROBE) reporting guideline for cohort studies.^16^

### Setting

World Trade Center responders are defined as individuals who worked or volunteered at disaster sites for 4 or more hours in the period from September 11 to 14, 2001; for 24 or more hours throughout September 2001; or for 80 or more hours between September 11, 2001, and July 1, 2002. The analytic sample is from a prospective cohort study of WTC responders enrolled in the Long Island, New York, WTC Health Program. Eligible responders were those who consented for data collected at monitoring appointments to be used in research. The program began enrolling responders in July 2002, immediately after the response efforts ended. The median year of enrollment was 2009 (IQR, 2005-2015), and the median time between visits was 12.6 months (IQR, 12.1-15.2 months).

Because lung cancer has a lengthy latency period, with estimates ranging from 20 to 34 years,^17^ we included only individuals who survived and were not lost to follow-up until at least 10 years after exposure completion (July 1, 2012). To facilitate follow-up, we included only individuals who were enrolled before July 1, 2022, to allow for sufficient time for at least 1 follow-up study by December 31, 2023, when data began being cleaned for analysis. Participants who developed lung cancer prior to program enrollment were excluded. A power simulation analysis^18^ indicated that a study with more than 100 cases of incident lung cancer would have high power (0.90) to identify a 1.75-fold increase in the incidence of lung cancer.

### Inclusion and Exclusion Criteria

Responders were fluent in English (>99% of responders listed English as their primary language), survived the collapse of the towers, and attended at least 2 monitoring visits from July 1, 2012, to December 31, 2023. Person-time accrued until a diagnosis of lung cancer (event). Participants were censored 2 years after the participant’s most recent study contact (administrative censoring for loss to follow-up), death from any cause other than lung cancer (censored), or at the end of the study period on December 31, 2023 (administrative censoring at the last data point available at the time of analysis). The number of participants lost to follow-up with or without a death reported were included, and cross-tabulations were used to evaluate their correlation with exposure.

### WTC Exposure Severity

Details about individuals’ exposures during work at the WTC site were collected at intake using an extensive questionnaire that was designed to ask about activities, exposures, and experiences during work at the WTC site. The questionnaire began to be administered immediately after exposures officially ended on July 1, 2002. Questions ranged from the things responders saw to activities they performed and protective equipment they wore.

Exposure activities examined in detailed analyses included dichotomous measures of place and duration of exposure in hours (eg, arrival on September 11, 2001; dust cloud exposure on September 11, 2001; worked on WTC sites in September 2001; ever worked on WTC sites; worked in enclosed spaces; worked on torch cutting; worked with concrete), sights (eg, human remains, bodily fluids, dust at worksite), smells (eg, mold, chemicals, fumes, sewage, smoke, diesel and nondiesel exhaust), other sensations (eg, noise, hot or cold temperatures), WTC-specific activities (eg, bucket brigade, sifting, firefighting, search or rescue, supervisorial work), hygiene (eg, wash hands to eat, dust mask use, respirator ownership and regularity of use, clothes changing, showering, Tyvek suit, hood suit), and general trades (eg, cable installation with or without manhole work, medical technician work, security, morgue, sanitation, engineering, canteen, or truck-related services including loading, escorting, driving, and routing).

Prior work has focused predominantly on using dust cloud exposure and arrival date as proxies for exposure severity.^13,19^ This approach has been criticized for ignoring the presence of settled dust as a potential inroad for exposure to fine particulates.^14^ Noting that concern, recent work developed a novel measure of fine particulate exposure that uses the activities-based approach to the exposure ranking already described.^20^ Because fine particulate exposures are invisible when aerosolized, this approach advocates using responders’ reported activities coupled with duration and timing of exposure to rank exposure severity into 5 groups ranging from minimal exposure (0) to severe exposure (4). In this study, the sample sizes in the lowest and highest severity groups were relatively small, so we collapsed the score into 3 categories including mild (originally, a score of 0 and 1), moderate (score of 2), and severe (score of 3 or 4). Sensitivity analyses were conducted using all 5 original categories.

### Lung Cancer Diagnosis

The date of lung cancer diagnosis was the outcome (*International Classification of Diseases, Ninth Revision* code 162 or *International Statistical Classification of Diseases and Related Health Problems, Tenth Revision* code C34). Diagnosis was completed using a 2-step certification process that was led by on-site clinical staff and later reviewed and verified by medically trained personnel at the Centers for Disease Control and Prevention. All patients in the WTC Health Program were screened for lung cancer according to the US Preventive Services Task Force recommendations.^21^ Chest radiography was completed biennially or when clinically indicated. An abnormality or nodule detected on a radiograph was further evaluated using computed tomography of the chest and followed the Fleischner Society guidelines for managing incidental pulmonary nodules.^22^ Pulmonary masses were evaluated by chest surgery for biopsy.

### Covariates

Demographic characteristics included the responder’s age in years centered at July 1, 2012, gender (male vs female), educational attainment (high school diploma or less, some college, a university degree or higher, or unknown), and self-reported race and ethnicity (Black, Hispanic, White, and other [American Indian or Alaska Native, Asian or Pacific Islander, multiracial, and race or ethnicity not otherwise specified]). Race and ethnicity were collected for demographic purposes. For descriptive purposes, we also reported responders’ occupation type at the time of exposure (categories including law enforcement, firefighter or emergency medical responder, military or federal responder, construction worker, and other). Exposures and pulmonary outcomes often differ between responders who were trained to be first responders and those who were not,^23^ so we report whether individuals were trained as responders. Information about smoking status was collected at monitoring visits and recorded using smoking pack-years at or after July 1, 2012, along with current self-reported smoking status (categorized as current, former, or never smoker).

Although not a central focus of the study, data on whether a prior exposure to WTC-like toxicants (eg, asbestos, cadmium, silica, fiberglass, lead, mercury, pesticides, noncigarette smoke, or solvents) during pre-WTC hobbies or at unrelated jobsites were also collected at enrollment. Responders ranked exposures from 0 (never or incidental) to 4 (daily), and both unique exposures and a mean of all reported exposures were used.

### Missing Data

The only missing data at baseline were data on individuals’ educational attainment. We addressed this by creating a category for individuals who did not report educational attainment so that their responses could be included (ie, a missing-indicator approach). Sensitivity analyses investigated the effect of imputing missing data on education for participants using an ordinal logistic model that included all study variables and found little effect, so we report results from the missing-indicator approach.

### Statistical Analysis

Sample characteristics on July 1, 2012, were described using mean (SD) values or frequencies and percentages. Nonparametric trend tests were used to compare differences in sample characteristics across levels of exposure severity. We calculated crude incidence rates (IRs) for the whole cohort and after stratification by exposure severity. We showed cumulative incidence curves, stratified by WTC exposure severity. The time scale used was the time, measured in days but expressed in years, after July 1, 2012—a decade after exposures had commenced and after the average responder had joined the monitoring program. Responders were censored either at the date of death from causes other than lung cancer or a decade after the beginning of the observational window (July 1, 2022). We then calculated risk differences, 95% CIs, and corresponding 2-tailed *P* values, with *P* < .05 considered significant. We used Cox proportional hazards regressions to estimate unadjusted and multivariable-adjusted hazard ratios (HRs).^24^ We used a robust Sandwich standard error, the Efron method for ties, and Schoenfeld residuals to assess the proportional hazards assumption. Multivariable-adjusted analyses accounted for demographics and smoking status and pack-years. We repeated Cox proportional hazards regressions for each exposure in the whole sample and when stratifying exposures by reported experiences, exposures, and activities on-site at the WTC. Results from models are recorded as multivariable-adjusted β coefficients from the Cox proportional hazards regression models and are reported using a heatmap. Cell borders were used to highlight statistical significance after adjusting for the false discovery rate using the Benjamini-Hochberg procedure (false discovery rate, 0.05).^25^ Analyses were completed using Stata, version 17/MP (StataCorp LLC).

In sensitivity analyses, we examined whether a history of exposures during hobbies or at work outside of the WTC sites were associated with lung cancer incidence. Fine-Gray competing hazards regression was used to evaluate the influence of mortality due to causes not associated with lung cancer as a source of competing risks. To consider the association of recall bias among individuals who were recruited later in the cohort, we compared the association of stratifying results or adjusting for year of enrollment and compared model estimates using *t* tests. To further examine associations between follow-up years and outcomes, we next stratified the study by the number of follow-ups that people attended. To consider possible sex differences in exposure dynamics, we conducted stratified analyses in males and females and compared the strength of associations.

## Results

**Figure 1.**
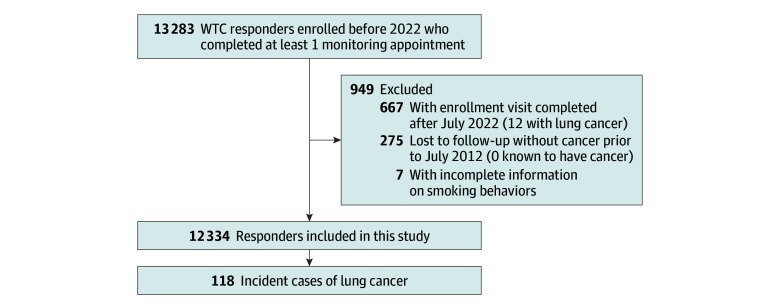
Study Flowchart Showing Number of Responders in Each Group

Of 13 283 responders residing on Long Island, New York, who participated in the monitoring program in the time frame of this study, 12 334 (92.9%; mean [SD] age at study inclusion, 49.3 [10.2] years; 11 213 men [90.9%] and 1121 women [9.1%]; 681 Black participants [5.5%], 535 Hispanic participants [4.3%], 10 404 White participants [84.4%], and 714 participants of other race or ethnicity [5.8%]) were eligible for this study (Figure 1 and Table 1). Attrition analyses showed that 1087 responders (8.8%) missed their last annual visit but were not listed as dead, while 544 responders (4.4%) died. Tabulations showed there was no association between those who were lost to follow-up and exposure severity, but there was an association between exposure severity and increased mortality.

**Table 1.** World Trade Center Responder Characteristics

Characteristic	Total population (N = 12 334)	Exposure		
		Mild (n = 6919)	Moderate (n = 4136)	Severe (n = 1279)
Age, mean (SD), y	49.3 (10.2)	48.4 (9.4)	50.6 (11.0)	50.1 (11.0)
Sex, No. (%)				
Female	1121 (9.1)	776 (11.2)	304 (7.4)	41 (3.2)
Male	11 213 (90.9)	6143 (88.8)	3832 (92.6)	1238 (96.8)
Smoking status, No. (%)				
Never smoker	8233 (66.8)	4886 (70.6)	2619 (63.3)	728 (56.9)
Former smoker	2426 (19.7)	1250 (18.1)	878 (21.2)	298 (23.3)
Current smoker	1675 (13.6)	783 (11.3)	639 (15.5)	253 (19.8)
Smoking status, mean (SD), pack-years	2.3 (8.3)	1.8 (6.9)	2.9 (10.0)	2.7 (9.3)
Educational attainment, No. (%)				
High school diploma	2171 (17.6)	825 (11.9)	932 (22.5)	414 (32.4)
Some college	6466 (52.4)	3854 (55.7)	2050 (49.6)	562 (43.9)
University degree	3063 (24.8)	2013 (29.1)	876 (21.2)	174 (13.6)
Less than high school	435 (3.5)	166 (2.4)	178 (4.3)	91 (7.1)
Unknown	199 (1.6)	61 (0.9)	101 (2.4)	38 (3.0)
Not trained as first responder^a^	4108 (33.3)	749 (10.8)	2362 (57.1)	997 (78.0)
Occupation during response efforts, No. (%)				
Law enforcement	7733 (62.7)	5859 (84.7)	1614 (39.0)	260 (20.3)
Firefighter or emergency medical services	274 (2.2)	187 (2.7)	74 (1.8)	13 (1.0)
Military and federal response	321 (2.6)	103 (1.5)	204 (4.9)	14 (1.1)
Construction	2747 (22.3)	419 (6.1)	1496 (36.2)	832 (65.1)
Other	1259 (10.2)	351 (5.1)	748 (18.1)	160 (12.5)
Race and ethnicity, No. (%)				
Black	681 (5.5)	405 (5.9)	209 (5.1)	67 (5.2)
Hispanic	535 (4.3)	285 (4.1)	195 (4.7)	55 (4.3)
White	10 404 (84.4)	5823 (84.2)	3501 (84.7)	1080 (84.4)
Other^b^	714 (5.8)	406 (5.9)	231 (6.0)	77 (6.0)

^a^ Responders with occupations (eg, electrician or news reporter) that do not train workers as first responders but who, nonetheless, participated in response efforts.

^b^ Includes American Indian or Alaska Native, Asian or Pacific Islander, multiracial, and race or ethnicity not otherwise specified.

Most responders (n = 6919) were placed in the minimal-mild exposure group (Table 1). Compared with responders who were mildly exposed, those who were more severely exposed were less likely to report female sex (776 of 6919 [11.2%] vs 41 of 1279 [3.2%]; *P* < .001) and more likely to report being a former or current smoker (2033 of 6919 [29.4%] vs 551 of 1279 [43.1%]; *P* < .001), have higher reported smoking pack-years (mean [SD], 1.8 [6.9] vs 2.7 [9.3]; *P* < .001), report not being trained as first responders (749 of 6919 [10.8%] vs 997 of 1279 [78.0%]; *P* < .001), and have lower educational attainment (university degree: 2014 of 6919 [29.1%] vs 17 of 1279 [13.6%]; *P* < .001).

### Incidence of Lung Cancer

**Figure 2.**
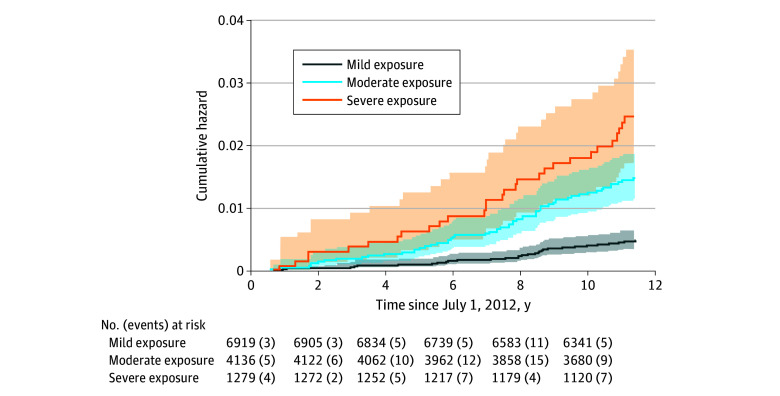
Cumulative Incidence of Lung Cancer at Least 10 Years After Response Efforts After the WTC Attacks Ended Shaded areas indicate 95% CIs.

During 135 009 person-years of follow-up, 118 cases of lung cancer were diagnosed (crude IR, 8.7/10 000 person-years [95% CI, 7.3-10.5/10 000 person-years]). Comparing the incidence of lung cancer across 3 exposure groupings identified an incremental increase in the incidence of lung cancer per 10 000 person-years. Cumulative incidence curves (Figure 2) identified an elevated incidence of lung cancer in the more severely exposed groups.

As shown in Table 2, the incidence of lung cancer was lowest (4.2/10 000 person-years [95% CI, 3.0-5.9/10 000 person-years]) in the mild exposure group vs 12.7 per 10 000 person-years (95% CI, 9.8-16.4/10 000 person-years) in the moderate exposure group and 21.0 per 10 000 person-years (95% CI, 14.6-30.3/10 000 person-years) in the severe exposure group. Participants in the moderate group (HR, 3.03 [95% CI, 1.97-4.67]; *P* < .001) and the severe exposure group (HR, 5.05 [95% CI, 3.05-8.34]; *P* < .001) had elevated hazards of lung cancer. Multivariable-adjusted models that further adjusted for demographics and smoking (Table 2; full results in the eTable in Supplement 1) reported attenuated but highly significant associations for moderate exposure (adjusted HR [AHR], 1.86 [95% CI, 1.19-2.91]; *P* = .007) and severe exposure (AHR, 2.90 [95% CI, 1.69-4.99]; *P* < .001).

**Table 2.** Incidence of Lung Cancer in World Trade Center Responders, Stratified by Exposure Severity

Exposure severity	Incident cases	No. of person-years of follow-up	Crude IR per 10 000 person-years (95% CI)	Risk difference per 10 000 person-years (95% CI)	*P* value	Unadjusted HR (95% CI)	*P* value	Multivariable AHR (95% CI)^a^	*P* value
Mild	32	76 238	4.20 (2.97-5.94)	0.00 [Reference]	NA	NA	NA	1.00 [Reference]	NA
Moderate	57	44 982	12.67 (9.77-16.43)	8.47 (4.88-12.06)	<.001	3.03 (1.97-4.67)	<.001	1.86 (1.19-2.91)	.007
Severe	29	13 789	21.03 (14.61-30.26)	16.83 (9.05-20.42)	<.001	5.05 (3.05-8.34)	<.001	2.90 (1.69-4.99)	<.001

Abbreviations: AHR, adjusted hazard ratio; HR, hazard ratio; IR, incidence rate.

^a^ Demographically adjusted hazards accounted for age, sex, educational attainment, smoking status, smoking pack-years, and responder training during the World Trade Center rescue and recovery events.

**Figure 3.**
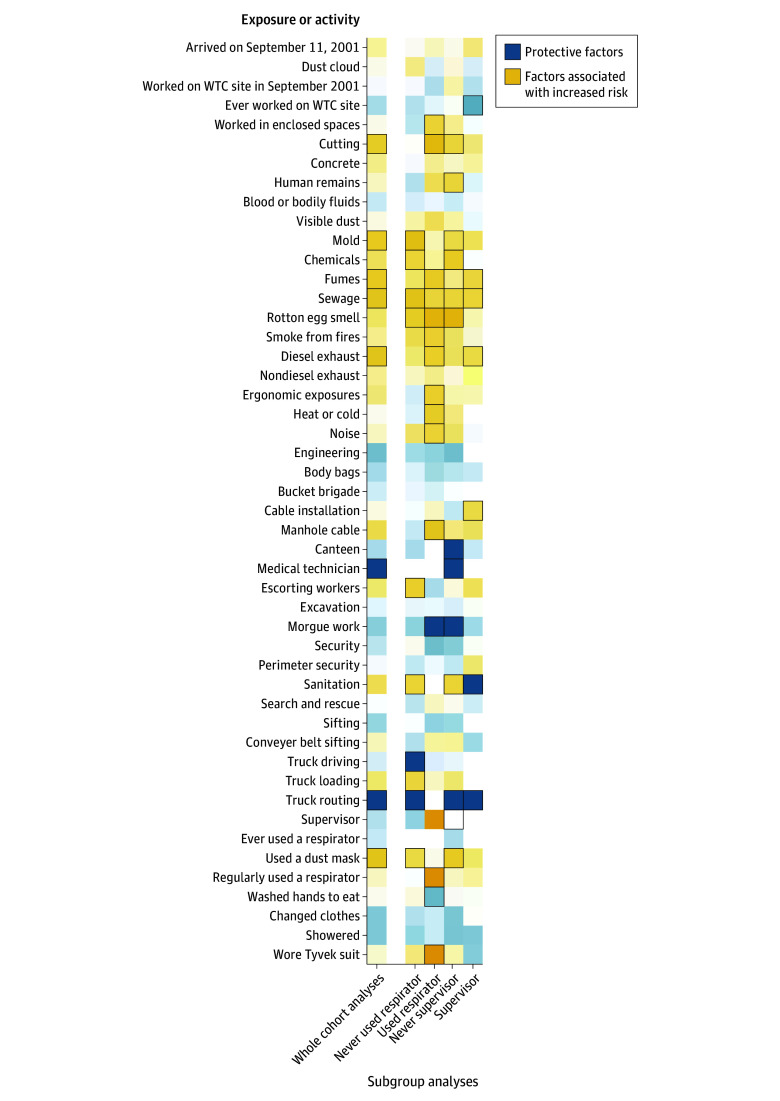
Heatmap Showing Multivariable-Adjusted Association Between Specific Exposure Activities at the World Trade Center (WTC) and the Incidence of Lung Cancer Results are shown first in the analytic cohort and then in subgroup analyses, after stratifying analyses based on whether responders ever wore respirators or acted as supervisors. Each cell shows results from a different regression model. Cells with black borders indicate statistically significant associations (2-tailed *P* < .05).

To clarify whether specific reported activities or exposures were associated with lung cancer, results were examined from survival models comparing exposures and incidence first in the full sample and stratified into groups by respirator ownership and supervisorial status as reported using a heatmap (Figure 3). There were several statistically significant results in these analyses that suggested that people who reported working in air that smelled moldy, smoky, or like chemicals was associated with an increased incidence of lung cancer. For example, working 500 or more hours in areas that smelled of sewage was associated with an increased risk of incident lung cancer (AHR, 1.06 [95% CI, 1.02-1.10]; *P* = .004). In contrast, working off-site, working in cleaner places, or performing nonexcavation work, such as working in the canteen, as well as several hygienic practices, such as changing clothes or wearing a hood suit, were associated with reduced incidence of lung cancer. The pattern of results in stratified analyses was consistent with the view that supervisors had smaller patterns of association across several exposures and with the incidence of lung cancer than those who worked as laborers.

Sensitivity analyses were completed to examine the association of changes in analytic choices. First, we examined the association of using a 3-group vs 5-group version of the exposure variable. The minimally exposed group had only 3 cases of lung cancer (IR, 2.5/10 000 person-years [95% CI, 0.8-7.9/10 000 person-years]), while the severely exposed group had 9 cases of lung cancer (IR, 14.3/10 000 person-years [95% CI, 7.5-27.5/10 000 person-years]). Group-based analyses found no differences between minimally exposed and mildly exposed groups but did identify an increase in the risk of incident lung cancer in the severely exposed group that appeared to be larger (HR, 5.92 [95% CI, 1.60-21.85]; *P* = .008) than in the highly exposed group (HR, 3.72 [95% CI, 1.11-12.52]; *P* = .03), but coefficients were not significantly different (*P* = .25), supporting the decision to combine small exposure groups. We examined associations with potential toxicants during hobbies or at other pre-9/11 worksites but found no statistically significant results before or after adjusting for confounders (solvent exposure: AHR, 1.01 [95% CI, 0.83-1.23]; lead exposure: AHR, 1.14 [95% CI, 0.90-1.45]). Next, we examined the use of competing hazards regression and found that the results were substantively similar to the results from Cox proportional hazards regression models (moderate exposure: AHR, 1.83 [95% CI, 1.16-2.86]; severe exposure: AHR, 3.06 [95% CI, 1.79-5.22]). Finally, we stratified the analysis based on enrollment year, number of monitoring visits completed, and sex, which did not change the interpretations and were not significantly different from one another (eFigure in Supplement 1).

## Discussion

We assessed activities-based exposure metrics and found that WTC site exposures were associated with lung cancer incidence 1 to 2 decades after the attacks. To our knowledge, this is the first study to characterize the association between WTC site exposure based on a severity index and lung cancer among responders, to examine activity-specific risk factors for lung cancer within a WTC site–exposed cohort, and to quantify the absolute and relative risk of lung cancer associated with WTC site exposures after a minimum 10-year latency period. Findings revealed that responders who were moderately and severely exposed exhibited a significantly higher hazard of developing lung cancer compared with those in the mild exposure group. In addition, this study replicated well-established associations by reporting that the incidence of lung cancer was higher among older responders and those who smoked or had lower educational attainment. The association between WTC site exposure severity and incident lung cancer remained appreciable and statistically significant after controlling for demographics, measures of tobacco smoke exposure, and educational attainment.

Our findings indicate that there is an increased rate of lung cancer among responders with greater WTC site exposure severity. We observed that the IR of lung cancer was 4.2 per 10 000 person-years among the mild exposure group, whereas the rate was comparatively higher among those with moderate exposure (12.7/10 000 person-years) and severe exposure (21.0/10 000 person-years). These results might seem contradictory when compared with previous work, where the incidence of lung cancer was lower among WTC responders than in the general population.^4,26^ However, we did not test, find, or report that lung cancer risk was different from the general population. In addition, our latency period is longer than other studies, which might benefit studies of lung cancer by allowing sufficient time for cases to accumulate.

Although not a central finding in this study, our study also supported a strong association between smoking and a higher incidence of lung cancer. Smoking is the most important risk factor for lung cancer in the general population^27,28^ and is among the most common risk factors used in clinical screening programs to determine risk of lung cancer among patients with lung nodules.^29^ Our results may support work to further reduce smoking behaviors in this population.

To our knowledge, this study is the first to demonstrate a strong association between WTC site exposure severity and lung cancer incidence, based on a severity score that incorporates probable exposure to fine particulate dust and other airborne chemicals from the WTC site.^15^ However, we also delved into the specific types of exposures associated with lung cancer and found that several experiential factors were associated with higher risk, including exposure to chemical smells (mold, fumes, and sewage). It is unlikely that such factors are independently associated with an increased risk of lung cancer. For example, other studies that focused specifically on exposure to airborne sulfur dioxide reported no association with risk of lung cancer.^30^ This finding could be due to the fact that people who work in dangerous places are more likely to be exposed to different chemicals and dust, making them more vulnerable to developing lung cancer, and recollection of sulfur smell is a proxy for such circumstances. Alternatively, it may be that individuals who remembered this smell did not use protective masks as effectively and were therefore more able to smell these odors while concurrently inhaling other less-odorous exposures.

Solan et al^31^ reported that WTC responders who worked off-site were less susceptible to developing all-cause cancer in the decade after response efforts when compared with those who worked at Ground Zero (the WTC sites). Our study also identified protective factors, including working in clean and nonexcavation locations or working off-site and maintaining hygienic practices, that could reduce the risk of cancer and may make respondents less vulnerable to developing lung cancer. This observation aligns with the findings of a study^32^ that similarly identified that individuals not engaged in recovery or rescue operations exhibited a lower likelihood of developing lung cancer compared with those actively involved in such roles. Together, results suggest that response activities that resulted in avoiding exposure to hazardous or contaminated environments may have played a protective role in reducing the risk of lung cancer.

### Limitations

This study has several important limitations. First, WTC exposure data were collected using a questionnaire that was not designed to measure the specific airborne dynamics of fine and ultrafine particulate matter, so exposure misclassification is possible. Second, the lack of biomarkers for WTC exposure severity forced us to rely on information collected retrospectively by responders at study intake. Although we did not find evidence of recall bias, exposure severity ratings could be improved by directly matching exposure questionnaires with biomarkers of exposure. In addition, while exposures are recorded, this study did not explicitly examine changes in behavior or the addition of other exposures occurring after WTC events that may induce additional biases. Third, we cannot exclude the possibility that exposures prior to the WTC event contributed to the risk of lung cancer. Sensitivity analyses comparing the number of visits, year recruited, and sex may help alleviate this concern, but we do not know the extent to which exposures prior to the WTC events are associated with exposure severity or the incidence of lung cancer. Fourth, we did not match data with regional cancer registries and therefore could not determine the degree of bias due to unobserved case events. However, prior work has established that cancer tends to be more reliably identified in this population than in the general population.^2^ This finding is generally attributed to the program’s ability to provide free evidence-based cancer screening, free treatment, and financial compensation to responders whose diagnoses of any cancer are reported to the monitoring program.

## Conclusions

Although increased risk of lung cancer is presumed in this population, this cohort study is the first to establish an association between a measure of WTC exposure severity and the incidence of lung cancer. Prior studies have shown a relatively low incidence of lung cancer among WTC responders, highlighting that a reliance on short latency periods along with lower smoking rates could result in lowered IRs.^2^ This study therefore underlines the importance of having an established and long-lasting monitoring program when the public is concerned about exposures whose outcomes are likely to have lengthy latency periods. This study establishes a need to begin to understand which specific carcinogens found at WTC sites might have increased the risk of lung cancer.

## Notes

Supplement 1. **eTable.** Full Adjusted Results From Cox Proportional Hazards Models Accounting for Demographics Including Pack-Years Smoking With and Without Current Smoking Status **eFigure.** Comparison of Hazards Ratios Estimated by Cox Proportional Hazards Regression for the Entire Sample (Leftmost Pair of Bars), and After Stratifying by Covariates Described in Sensitivity Analyses, and Within Specific Subgroups

Supplement 2.
Data Sharing Statement
