# Errors in Table 2

**DOI:** 10.1001/jamanetworkopen.2025.51437

**Published:** 2025-12-10

In the Original Investigation titled “Lung Cancer Incidence After September 11, 2001, Among World Trade Center Responders,”^1^ published October 9, 2025, there were errors in the fifth column of Table 2. The risk differences per 10 000 person-years for lung cancer should be 0.00 [Reference] for mild exposure, 8.47 (95% CI, 4.88-12.06) for moderate exposure, and 16.83 (95% CI, 9.05-20.42) for severe exposure. This article has been corrected.^1^
