# Supplementary material for: Lung Cancer Incidence After September 11, 2001, Among World Trade Center Responders
Source: JAMA Netw Open. 2025 Oct 9;8(10):e2536655. doi: 10.1001/jamanetworkopen.2025.36655 (PMC12511991; doi:10.1001/jamanetworkopen.2025.36655)
Supplement: Supplement 1. — eTable. Full Adjusted Results From Cox Proportional Hazards Models Accounting for Demographics Including Pack-Years Smoking With and Without Current Smoking Status eFigure. Comparison of Hazards Ratios Estimated by Cox Proportional Hazards Regression for the Entire Sample (Leftmost Pair of Bars), and After Stratifying by Covariates Described in Sensitivity Analyses, and Within Specific Subgroups [file jamanetwopen-e2536655-s001.pdf]

Supplementary Online Content

Clouston SAP, Meliker J, Mann FD, et al. Lung cancer incidence after September 11, 2001, among World Trade Center responders. *JAMA Netw Open.* 2025;8(10):e2536655. doi:10.1001/jamanetworkopen.2025.36655

eTable. Full Adjusted Results From Cox Proportional Hazards Models Accounting for Demographics Including Pack-Years Smoking With and Without Current Smoking Status
eFigure. Comparison of Hazards Ratios Estimated by Cox Proportional Hazards Regression for the Entire Sample (Leftmost Pair of Bars), and After Stratifying by Covariates Described in Sensitivity Analyses, and Within Specific Subgroups

This supplementary material has been provided by the authors to give readers additional information about their work.

© 2025 Clouston SAP et al. *JAMA Network Open.*

eTable. Full Adjusted Results From Cox Proportional Hazards Models Accounting for Demographics Including Pack-Years Smoking With and Without Current Smoking Status

Participant Characteristic	Adjusting for Demographics and Smoking Pack-Years aHR (95% C.I.)	P-Value	Additionally adjusting for Current Smoking Status aHR (95% C.I.)	P-Value
**WTC Exposure Severity**				
Mild	1.00		1.00	
Moderate	1.83 (1.16-2.86)	0.009	1.86 (1.19-2.91)	0.007
Severe	3.03 (1.77-5.18)	<0.001	2.90 (1.69-4.99)	<0.001
Age, years	1.08 (1.07-1.10)	<0.001	1.08 (1.06-1.09)	<0.001
Female Sex	1.79 (0.92-3.48)	0.088	1.65 (0.84-3.25)	0.145
Smoking, Pack-Years	1.02 (1.01-1.03)	<0.001	1.01 (1.00-1.02)	0.142
Smoking Status				
Never Smoker			1.00	
Former Smoker			3.88 (2.26-6.69)	<0.001
Current Smoker			6.64 (3.93-11.23)	<0.001
Educational Attainment				
High School Diploma	1.00		1.00	
Some College	0.51 (0.33-0.80)	0.003	0.55 (0.35-0.85)	0.007
University Degree	0.39 (0.19-0.77)	0.007	0.48 (0.24-0.95)	0.036
Less than High School	2.41 (1.39-4.19)	0.002	2.08 (1.20-3.58)	0.009
Unknown	0.69 (0.29-1.68)	0.418	0.69 (0.28-1.67)	0.410
Trained as first responder	1.07 (0.69-1.66)	0.756	0.96 (0.62-1.48)	0.844

Note: aHR: multivariable adjusted hazards ratio; 95% CI: 95% confidence interval. [a] The only difference between the two models is whether we adjusted for the current reported smoking status.

© 2025 Clouston SAP et al. *JAMA Network Open.*

eFigure. Comparison of Hazards Ratios Estimated by Cox Proportional Hazards Regression for the Entire Sample (Leftmost Pair of Bars), and After Stratifying by Covariates Described in Sensitivity Analyses, and Within Specific Subgroups as Marked Using "*". Gold bars show Hazards Ratios from Cox models for severe exposures, while turquoise blue striped bars showing Hazards Ratios for moderate exposures; 95% confidence intervals are shown using error bars. None of the coefficient estimates are statistically significantly different from the overall unadjusted estimate (leftmost bar; p-values range from 0.12-0.99).

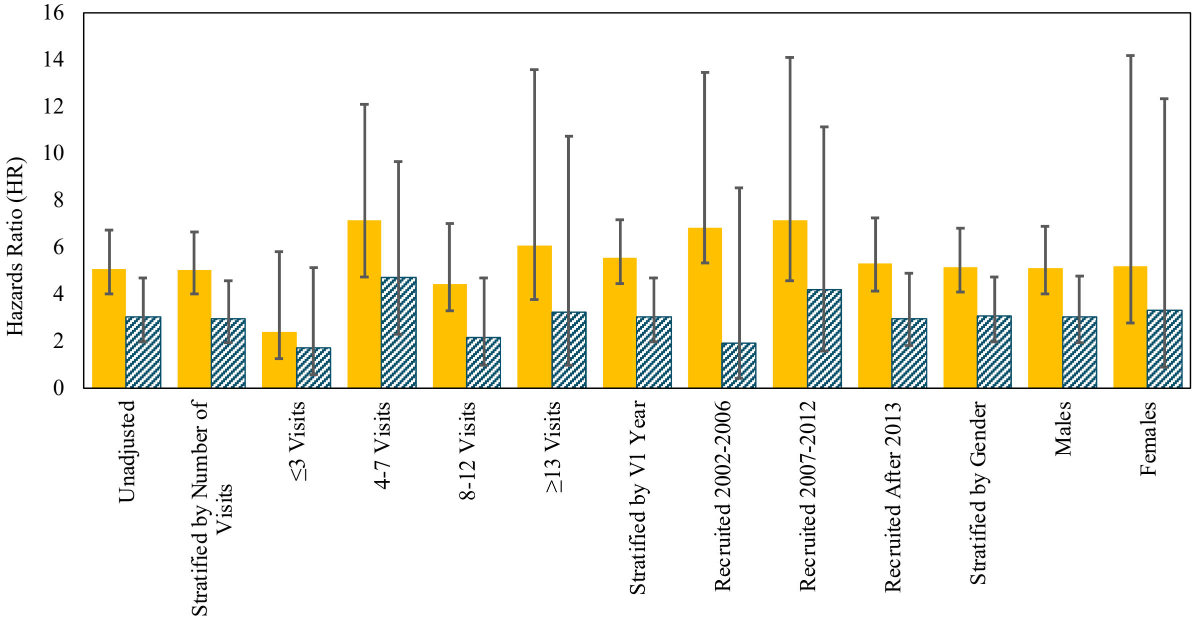

© 2025 Clouston SAP et al. *JAMA Network Open.*
