# Supplementary material for: Lung Cancer Incidence After September 11, 2001, Among World Trade Center Responders
Source: JAMA Netw Open. 2025 Oct 9;8(10):e2536655. doi: 10.1001/jamanetworkopen.2025.36655 (PMC12511991; doi:10.1001/jamanetworkopen.2025.36655)
Supplement: Supplement 2. — Data Sharing Statement [file jamanetwopen-e2536655-s002.pdf]

Data Sharing Statement

Clouston. Lung Cancer Incidence After September 11, 2001, Among World Trade Center Responders. *JAMA Netw Open*. Published October 09, 2025.
doi:10.1001/jamanetworkopen.2025.36655

Data

Data available: Yes
Data types: Deidentified participant data
How to access data: https://osf.io/hxye6/
When available: With publication

Supporting Documents

Document types: Statistical/analytic code
How to access documents: https://osf.io/hxye6/
When available: With publication

Additional Information

Who can access the data: Researchers whose use of the data has been approved.
Types of analyses: Any specified purpose.
Mechanisms of data availability: After a signed data access agreement.
